# Validation of the Edinburgh Postnatal Depression Scale against both DSM-5 and ICD-10 diagnostic criteria for depression

**DOI:** 10.1186/s12888-018-1965-7

**Published:** 2018-12-20

**Authors:** Johanne Smith-Nielsen, Stephen Matthey, Theis Lange, Mette Skovgaard Væver

**Affiliations:** 1 Centre for Early Intervention and Family Studies, Department of Psychology, University of Copenhagen, Oester Farimagsgade 2A, DK-1353 Copenhagen, Denmark; 2 South West Sydney Local Health District, Liverpool Hospital, University of Sydney, Sydney, Australia; 3 Department of Public Health, Section of Biostatistics, University of Copenhagen, Copenhagen, Denmark

**Keywords:** Depression, Postnatal, Two-phase design, Screening, Exploratory factor analysis, Perinatal anxiety

## Abstract

**Background:**

The Edinburgh Postnatal Depression Scale (EPDS) is widely used in many countries to screen women for depression in the perinatal period. However, across studies the psychometric properties and cutoff scores of the EPDS have varied considerably; potentially due to different depression criteria and diagnostic systems being used. Therefore, we validated the Danish EPDS against a depression diagnosis according to both DSM-5 and ICD-10. Furthermore, we examined whether the Danish EPDS is multidimensional, as it has previously been suggested.

**Methods:**

Women (*N* = 324) were recruited after routine screenings with the EPDS between 2 and 10 months postpartum (T1). At a subsequent home visit (T2), the EPDS and the Structured Clinical Interview for DSM-5 were administered. Diagnostic interviews were audio recorded to enable subsequent coding for ICD-10 diagnoses and inter-rater reliability analysis. A two-phase stratified sampling strategy with three sampling categories (EPDS-score at T1) was used. Using the distribution of 4931 T1 EPDS-scores from the same population from which we sampled the participants, we used sampling weighing to reweight the sample. The calculation of weights was based upon the mother’s sampling category at T1 (i.e. the probability of being sampled) and the weights were applied when assessing the receiver operation characteristics (ROCs) of the EPDS. Sensitivity, specificity, positive predictive value, negative predictive value and area under the ROC curve were computed from the reweighted data for all relevant cutoff values. CIs were computed by embedding the calculations in a weighted logistic regression. Exploratory factor analysis was done using oblique rotation. Parallel analysis was used to assess the number of factors.

**Results:**

A score of 11 or more was found to be the optimal cutoff for depression according to both DSM-5 and ICD-10 criteria. Factor analysis suggested that the Danish EPDS consists of three factors, including an ‘anxiety factor’.

**Conclusions:**

The Danish EPDS has reasonable sensitivity and specificity at a cutoff score of 11 or more. There are no notable differences with respect to using ICD-10 or DSM-5 criteria for depression in terms of optimal cutoff. The variation in cutoff scores is likely to be due to cultural variations in the expression of depressive symptoms.

**Electronic supplementary material:**

The online version of this article (10.1186/s12888-018-1965-7) contains supplementary material, which is available to authorized users.

## Background

The Edinburgh Postnatal depression Scale (EPDS) [1] has been established as a useful screening instrument for detection of women at risk for depression in the perinatal period [2, 3]. In addition, it has been identified as the most frequently validated instrument to screen for perinatal depression [4], and in 2014 the EPDS had been validated against a diagnosis of depression in at least 37 languages [5]. Although recently, a discussion has begun to emerge regarding several shortcomings in the scale, including the multitude of different validated cutoff scores required for women (and men) from different cultures and during the ante- and postnatal periods, as well as the exclusion of certain types of psychological distress often occurring in the perinatal period (e.g. perinatal anxiety) [6], the current use of the EPDS in Denmark warrants understanding its properties further.

For almost two decades, a translation of the EPDS [7] has been in used for screening purposes in primary care in Denmark by public health visitors, and this translation was also used in a large-scale study to assess risk factors and point prevalence of postnatal depression in Danish women [7]. Nevertheless, the Danish EPDS has not been validated in a Danish population, and no official guidelines exist regarding cutoff scores for further monitoring of symptoms or referral to other services. This has resulted in various cutoff scores being used across the public health visiting districts where the scale is most frequently used. Moreover, the scale has only rarely been used in general practice or as part of perinatal psychiatric services where it is required that a screening instrument must go through a formal validation before implemented in practice.

The most commonly used cutoff score in postnatal women is 13 or more [8]. However, across studies, and in particular across languages, the optimal cutoff scores of the EPDS have varied considerably [5]. For example, the optimal cutoff for a diagnosis of depression in postnatal woman was found to be 7 or more in a Lithuanian population [9], 9 or more in a Sinhala population [10], 11 or more in a French population [11], and 12 or more in a Swedish population [12]. These differences in identified cutoff scores may be due to cultural variation in the expression of depressive symptoms in the perinatal period [13, 14], and consequently, researchers stress that the EPDS should be validated in a particular population before implementation in screening programs [15].

Another reason for the differing identified cutoff scores may be that across studies different diagnostic criteria and different diagnostic systems have been used [16]. Some, (e.g. [17]) have used criteria for depression according to their current version of the Diagnostic and Statistical Manual for Mental disorders (DSM–III–R; IV–TR; 5) [18, 19]. Others, (e.g. [10]) have used the International Classification of Diseases 10th revision (ICD-10) [20]. The original and some subsequent early validation studies [1, 21] used the Research Diagnostic Criteria (RDC) [22]. Differences between the diagnostic systems, with respect to diagnoses of depression, are marked. Whereas DSM-IV allows for a diagnosis of minor depression, this diagnosis does not exist in DSM-III, DSM-5, or ICD-10. Some studies have reported the optimal cutoff score for detecting just DSM major depression [12], while others have reported the optimal cutoff score for minor *and* major depression, sometimes referred to as ‘combined depression’ [23, 24]. Yet other studies have reported separate cutoffs, i.e. for minor or major as well as for just major depression [25]. Yet another difference between the ICD and DSM is that ICD-10 requires a minimum of two out of three core symptoms (depressed mood, anhedonia, and energy loss) for a diagnosis of depression, whereas DSM (III, IV, and 5) only requires the presence of one of two core symptoms (depressed mood and anhedonia). Moreover, none of the ICD-10 diagnoses of mild, moderate, or severe depression correspond to DSM-IV minor or DSM-III/IV/5 major depression in terms of symptom requirements (ICD-10 mild: 2 core symptoms + 2 or 3 associated symptoms; ICD-10 moderate: 2 core symptoms + 4 or 5 associated symptoms; ICD-10 severe: 3 core symptoms + 5–7 associated symptoms. DSM-IV minor: at least 1 core symptom + 2–4 symptoms; DSM-III/ IV/ 5 major: at least 1 core symptom + 5–9 symptoms). Similar discrepancies occur in the RDC diagnostic system in which a diagnosis of major depression requires at least one core symptom and at least five associated symptoms [22]. It is not, known whether ICD-11 which, if endorsed by member states, is planned to come into use in 2022, will be any different in these respects from ICD-10. Finally, some diagnoses have been made determining if criteria are met using in-depth diagnostic interviews, such as the Structured Clinical Interview for DSM-IV axis I disorders [26] which allows for probing and further exploration of answers, i.e. to determine if a symptom is in fact present, while others have used interviews that do not allow such probing, e.g., the Mini-International Neuropsychiatric Interview [27].

All of these issues could account for some of the differences in optimal cutoff scores reported across EPDS studies, notwithstanding the language or cultural differences [16]. As yet, however, no study has investigated whether using different diagnostic systems influence receiver operating characteristics of the EPDS. Therefore, we validate the EPDS against both ICD-10 and DSM-5. Whilst the DSM is most frequently used in EPDS validation studies, and in research more generally [28], the ICD is the official coding system in most of the countries where the EPDS is being used for screening purposes. Indeed, the ICD-10 has been identified as the most frequently used in clinical practice across countries [29], and hence, from a clinical perspective, it is relevant to include ICD criteria in a validation of the EPDS.

Apart from the EPDS’s utility in screening for depression, several investigators [30, 31] have commented on its properties for screening for perinatal anxiety given that this mood disorder is also prevalent, often co-occurs with depression, and has significant impacts not only on the mother’s well-being but also on her offspring [32, 33]. Although the EPDS was intended to be unidimensional, using exploratory factor analysis (EFA), many studies have suggested that the scale contains two factors, a depressive factor and an anxiety factor (e.g. [11, 34, 35]). Other studies have suggested that a three-factor structure with depression, anxiety, and anhedonia (e.g. [36, 37]), or depression, anxiety, and self-harm/suicide (e.g. [38, 39]) fits the data better. There is also variation as to which items load on the anxiety scale. While the anxiety factor most frequently include items 3 (guilt),4 (anxiety),and 5 (panic attacks) [40], some studies have found other item combinations to comprise the anxiety factor, e.g. Italian version: items 4, 5 & 6 [41]; Iranian version: items 3,4,5 and 8 [42]. More recently, a researchers have questioned the utility of continously conducting EFA as opposed to data and theory-driven confirmatory factor analyses (CFAs), (e.g. [40, 43]) and related to this, the optimal length of the EPDS has been questioned [44]. However, as there is currently no genereally agreed upon factor structure that could serve as the basis for a CFA, in the present study, we decided to assesss the factor structure of the EPDS using EFA.

### Aims of the study

To address these issues, the aims of the present study were to a) validate the Danish version of the EPDS against a diagnosis of depression in a sample of new mothers by assessing the sensitivity, specificity, and predictive values of different cutoff scores; b) investigate whether these receiver operating characteristics (ROCs) of the scale differ depending upon whether the DSM-5 or the ICD-10 is used, and (3) using an exploratory factor analytic approach, examine the factor structure of the Danish EPDS.

As ICD-10 mild depression in some contexts is considered to be subthreshold [45], for comparison, we conducted ROC analyses with and without including ICD-10 mild depression as ‘depressed’. In the determination of the optimal cutoff, for first-phase screening purposes, we intended to select a value that provides good sensitivity (the true-positive rate) and high specificity (the true-negative rate) without lowering the positive predictive value (PPV: proportion of subjects with positive test results who are correctly diagnosed) so much that it would overwhelm clinical services. Based on the view that a missed case of depression in the postnatal period can have significant negative consequences, we aimed at a sensitivity of 80% or more, a specificity of 90% or more, and a positive predictive value of 50% or more.

## Methods

### Study setting and procedure

As part of the general social security and health care system in Denmark, all families in are offered health visits by public health visitors (specialized nurses) in their home during the first year postpartum. This study was conducted in collaboration with the health visitors from the municipality of Copenhagen and was part of a larger project, the Copenhagen Infant Mental Health Project (CIMHP), which also includes a treatment trial (Clinical trials identifier: NCT02497677) [46]. Enrollment of participants started July 2015 and data collection for the present study terminated June 2017.

During the project period, all mothers in Copenhagen received home visits at 2 and 8 months postpartum by public health visitors. First-time mothers received an additional visit at 4 months postpartum. The EPDS was routinely administered at the two month visit, however, in addition, some women were also administered the EPDS at 4 or 8 months based upon the clinical judgement of the health visitor. This score is the Time 1 (T1) EPDS score. To ensure that sufficient numbers of women who met criteria for depression were recruited, an oversampling strategy was used, similar to that used in other studies [17, 21, 47]. Therefore, all mothers scoring 10 or more at T1 were invited to participate in CIMHP. Additionally, a subgroup of health visitors, equally distributed across districts, invited (from April 2016 – February 2017) not only mothers scoring 10 or more, but also those who scored 0–9 at the routine two month visit to the project. The sampling strategy is described in further detail below.

After screening with the EPDS, the health visitor informed the mother about the research project, and if interested, contact information was given to the research team. Interested mothers were offered a home visit by a clinical psychologist from CIMHP (Time 2: T2). During this visit, written informed consent was obtained, the EPDS was again administered, and a diagnostic interview was conducted. For logistic and practical reasons, the time-period from T1 to T2 could vary from a few days to several weeks. Therefore, to obtain the most precise ROC estimates, the EPDS score obtained at the T2 was validated in the current study.

### Sampling strategy and weighting

Mothers were eligible for participation in the current study if they were at least 18 years old, if they had an infant between 8 weeks and 10 months, and if they could read and speak Danish. We used a two-phase stratified sampling design [48] also used in other validation studies e.g. [17, 49]. A data extraction from the health visitors’ digital filing system (14 February 2016) including the latest 4931 EPDS-screenings (i.e. T1 scores) from mothers with infants under one year in Copenhagen showed that 69% of all screened mothers scored in the range 0–5, and 21% scored in the range 6–9, and 10% scored 10 or more. Thus, the vast majority were expected to score less than 6. In order to enrich the sample in the range where the cutoff was expected to be found, and to ensure roughly equal representation of these groups, we included all mothers scoring 10 or above (probable cases) at T1, and a larger proportion of those in the range 6–9 (possible cases) than of those scoring in range 0–5 (probable non-cases). The aim was to include at least 35 mothers from each of the two lower groups. To obtain these goals after October 2016 we only invited mothers who scored 6–9 or 10 or more at T1. The 4931 scores effectively gave us the population wide distribution and we could therefore use sampling weighing to reweight the sample corresponding to if we had done a random sample from the full population. The calculation of weights was based upon the mother’s sampling category at T1 (i.e. the probability of being sampled), and the weights were subsequently applied to the ROC analyses of T2 EPDS scores. Because all mothers scoring 10 or above were included, this group had the weight 1. The 0–5 and 6–9 groups got the sampling weights 28.8 and 16.9, respectively. By construction, after applying these weights, the distribution of T1 scores in our weighted sample matched the one observed in the full population, and as no other systematic effects of the sampling procedure exists, the weighted analysis can therefore be thought of as a simple random sample from the full population, but with substantial higher statistical power compared to a true random sample from the full population. This mimics well known techniques from survey literature (see [50]).

### Measures

The Edinburgh Postnatal Depression Scale [1] is a 10-item self-report questionnaire (range 0–30) designed to screen for possible depression in new mothers, and was completed by the mothers at T1 and T2. While the original published version of the Danish translation [7] had some formatting differences to the English version (i.e. inclusion of response scores; altering the item wording, and exclusion of the introduction), these were amended so that the Danish version used in the study was identical to the English version, having gone through the usual translation and back-translation methodology and complied with the principles for translation of the EPDS described in the EPDS manual [5]. The Danish EPDS is provided as Additional files 1 and 2.

The Structured Clinical Interview for the DSM-5 (SCID-5) [51] was used at T2 to establish a diagnosis of major depression according to DSM-5 as well as to assess history of depression. The interviewers were trained to explore the mothers’ answers to the standardized questions to be able to differentiate between normal and depressive reactions in the postnatal period (such as sleep problems and changes in appetite) because it has been found that depression might be otherwise over diagnosed in new mothers [52]. The interviews were conducted by trained SCID-5 interviewers who received ongoing supervision, and the interviews were audiotaped to allow for inter-rater reliability analyses to be conducted. The SCID-5 scorings and audio recordings of the interviews were also used to diagnose mothers according to ICD-10 diagnostic criteria for depression (mild, moderate, and severe depression). The interviewers did not score the T2 EPDS filled in by the mother prior to the interview. However, it was not possible to blind the interviewers entirely to the T1 or T2 EPDS scores (e.g., sometimes, the mother mentioned her T1 score prior to or during the interview). Therefore, to prevent interviewer-biases and ensure interrater reliability, a randomly selected subset (*n* = 70*,* 22%) of the audio recorded interviews were rated by a certified SCID-5 interviewer. This rater had no previous knowledge about the mothers and was blind to EPDS score and the diagnoses made by the interviewers. Interrater agreement for DSM-5 diagnostic status (no depression vs. major) was 90.2%, *κ* = .89 (*p* = .000); for ICD-10 diagnostic status (no depression vs. mild or more) interrater agreement was 94.6%, *κ* = .94 (*p* = .000), and for ICD-10 diagnostic status, four-way, interrater agreement was 94.6% (no depression); 81.8% (Mild), 78,6%; (Moderate), and 80% (Severe), *κ* = .76, (*p* = .000) which are all considered to represent excellent levels of interrater reliability [53].

### Statistical methods

Sensitivity, specificity, positive predictive value (PPV), negative predictive value (NPV) and area under the ROC curve (AUC) were computed directly from the reweighted data and the calculations were done for all relevant cutoff values. Confidence intervals were computed by embedding the calculations in a weighted logistic regression as implemented in R version 3.3.1. Confidence intervals corrected for weights were then obtained. Exploratory factor analysis (EFA) was done in R using the Psych Package [54]. To allow for factors’ inter-correlation, as would be expected of the underlying assumed dimensions of depression, anhedonia, and anxiety [55] we employed oblique rotation (promax). We used parallel analysis, i.e. using scree plots comparing actual loadings with random data with the same properties as the real data, to assess the appropriate number of factors. Continuous variables were summarized using means and standard deviations while categorical variables were summarized using raw counts and percentages.

## Results

A total of 350 mothers agreed to be contacted by the research team after T1 screening. Of these, 23 declined to participate when contacted, 2 did not consent to participate after having received a home visit from the research team, and one mother was not able to fill in the EPDS in Danish, and was therefore excluded, resulting in a final sample size of 324 women for whom we had T2 EPDS scores and a diagnostic interview. For four of these mothers, the T1 EPDS score was not available, and therefore data from these mothers were only included in unweighted analyses and not in the weighted analyses for which *n* = 320.

**Fig. 1:**
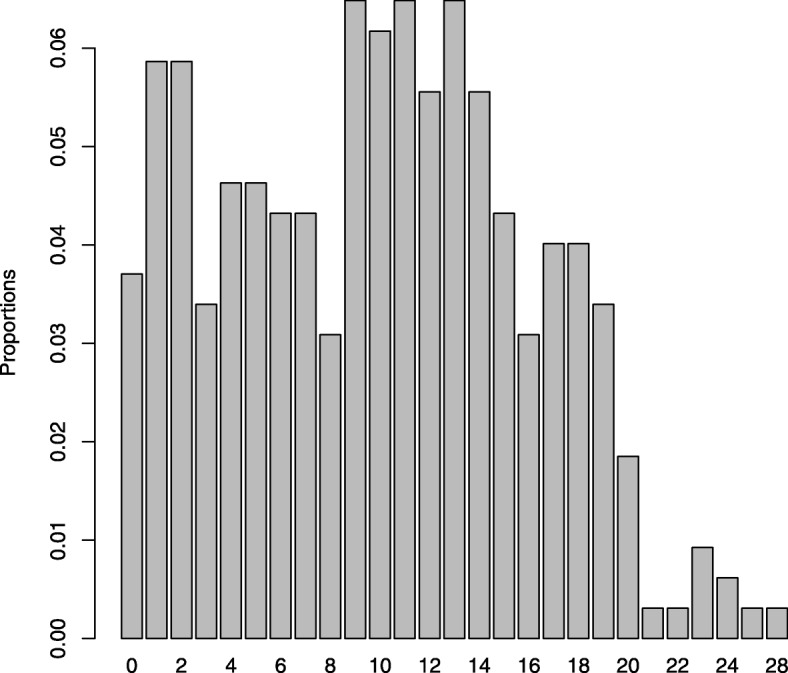
EPDS scores (T2 raw counts)

**Fig. 2:**
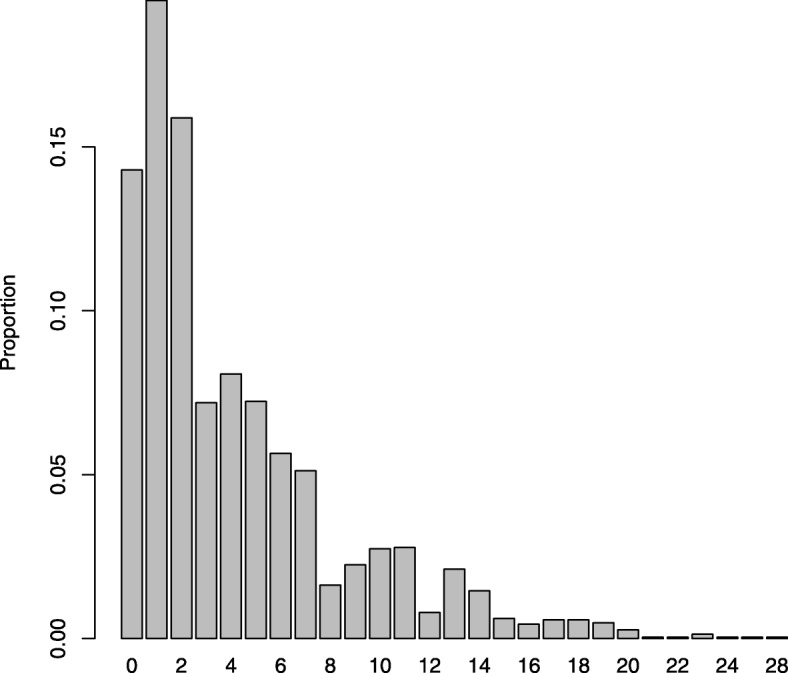
EPDS scores (T2 weighted)

The distribution of mothers across the three sampling categories at T1 were as following: EPDS range 0–5: *n* = 56 (17.5%), EPDS range 6–9: *n* = 29 (9.1%), and EPDS range 10–30: *n* = 235 (73.4%). Thus, the desired size of 35 mothers from the each of the two lowest sampling groups (at T1) was achieved for the lowest group and almost for the middle group. The distribution of raw EPDS scores at T2 is presented in Fig. 1. The unusual distribution is due to the sampling mechanism. The reweighted distribution, reflecting the distribution in the population, i.e. based on the distribution of the latest 4931 EPDS screenings in Copenhagen, is presented in Fig. 2.

Sample characteristics and distribution across diagnostic categories are presented in Table 1. As shown in Table 1, the majority (83%) of the mothers had an infant between 8 and 20 weeks at T2 when the diagnostic interview was conducted. Non-weighted EPDS means for the diagnostic categories were: DSM-5 Major: *M* = 15.0 (*SD* = 4.5)*,* not depressed: 7.0 (*SD* = 4.5); ICD-10 mild: *M* = 12.5 (*SD* = 4.0), ICD-10 moderate: *M* = 14.9 (*SD* = 4.2), ICD-10 severe: 18.4 (*SD* = 3.8), not depressed: *M* = 7.1 (*SD* = 4.9).

**Table 1:** Sample characteristics and distribution of diagnostic categories

Variable	*N* = 324	
Maternal age, mean (*SD*)	32.7	(4.6)
Range	22–45	
Infant age^a^ in weeks, mean (*SD*)	16.2	(8.2)
Infant age 8–14 weeks, *n* (%)	211	(65.1)
Infant age 15–20 weeks, *n* (%)	58	(17.9)
Infant age 21–30 weeks, *n* (%)	30	(9.3)
Infant age 31–49 weeks, *n* (%)	25	(7.7)
Infant gender, boys, *n* (%)	174	(53.7)
Maternal country of origin		
Danish, *n* (%)	271	(83.6)
Immigrant, *n* (%)	32	(9.9)
Descendants of immigrants, *n* (%)	9	(2.8)
Missing, *n* (%)	12	(3.7)
Mother single parent or not		
Married or living with partner, *n* (%)	293	(90.4)
Single, *n* (%)	14	(4.3)
Other, *n* (%)	6	(1.9)
Missing, *n* (%)	11	(3.4)
Primiparous *n* (%)	192	(63.6)
Maternal ISCED level of education		
Level 1–3 (lower secondary or less), *n* (%)	29	(9.0)
Level 4 and 5 (post secondary + short-cycle tertiary), *n* (%)	37	(11.4)
Level 6 (bachelor or equivalent), *n* (%)	113	(34.9)
Level 7 and 8 (master + doctor or equivalent), *n* (%)	134	(41.4)
Other or missing, *n* (%)	11	(3.4)
Mothers endorsing item 10 above zero, *n* (%)	26	(8.0)
Depression history status^b^		
No current or past depression, *n* (%)	132	(40.7)
Not currently depressed but a history of depression, *n* (%)	75	(23.1)
Currently depressed with a history of depression, *n* (%)	62	(19.1)
First episode of depression, *n* (%)	55	(17.0)
DSM-5 Major depression^c^		
Depressed, *n* (%)	118	(36.4)
Not depressed, *n* (%)	206	(63.6)
ICD-10 any (mild, moderate, or severe depression)^c^		
Depressed *n* (%)	120	(37.0)
Not depressed, *n* (%)	204	(63.0)
ICD-10 moderate or severe depression^c^		
Depressed *n* (%)	82	(25.3)
Not depressed, *n* (%)	242	(74.7)

*Note.* ISCED = International Standard Classification of Education (UNESCO). ICD-10 = International Classification of mental and behavioural disorders 10th edition (WHO, 1992); DSM-5 = Diagnostic and Statistical Manual of Mental Disorders, 5th edition (American Psychiatric Association, 2013)

^a^Infant age at the day for diagnostic interview and EPDS screening (T2)

^b^History of depression status was established according to DSM-5 criteria

^c^Due to the oversampling strategy, these are not prevalence estimates. See text

Table 2 shows the overlap between the two diagnostic systems. As shown, 10 mothers had a discrepant diagnostic status: four of the 118 mothers fulfilling criteria for DSM-5 major depression, did not fulfill criteria for any ICD-10 diagnosis. Inspecting the data further showed that these mothers only had one core symptom (but five symptoms in total), and hence, they did not get a ICD-10 diagnosis. Of the 38 mothers fulfilling criteria for ICD-10 mild depression, six did not fulfill criteria for DSM major depression. These mothers all had four symptoms (two core and two associated symptoms), thereby lacking one symptom to fulfill criteria for DSM-5 major depression but had the lowest possible number of symptoms to fulfill criteria for ICD-10 mild depression.

**Table 2:** Cross tabulation of distributions of ICD-10 and DSM-5 diagnoses

	DSM-5 diagnostic status		
ICD-10 diagnostic status	No depression	Major depression	Total
No depression	200	4	204
Mild depression	6	32	38
Moderate depression	0	60	60
Severe depression	0	22	22
Total	206	118	324

*Note.* ICD-10 = International Classification of mental and behavioural disorders 10th edition (WHO, 1992); DSM-5 = Diagnostic and Statistical Manual of Mental Disorders, 5th edition (American Psychiatric Association, 2013)

Cronbach’s alpha, a measure of a scale’s reliability, was 0.822 in the raw data, and in the weighted data it was 0.835, indicating good internal consistency [56].

### Receiver operating characteristics

Tables 3 presents estimates of sensitivity, specificity, PPV, NPV, and AUC for DSM Major depression, ICD-10 mild depression or more, and ICD-10 moderate+severe depression for different EPDS cutoff-scores and based on the reweighted data. For all three diagnostic categories of depression, the AUC values were close to 1 (ranging from 0.957–0.960) indicating that the Danish EPDS has a high discriminative power (i.e. how well the test separates the group being tested into those with and without the condition).

**Table 3:** Sensitivity, specificity, PPV, NPV, and AUC for DSM-5 major depression, ICD-10 mild, moderate, and severe depression, and ICD-10 moderate and severe depression

EPDS cutoff^a^	Sensitivity	95% CI	Specificity	95% CI	PPV	95% CI	NPV	95% CI	AUC
DSM-5 major									
6 + ^b^	98.0		77.0		22.4		99.8		0.960
7+	96.6		83.0		27.9		99.7		
8+	84.6		87.7		31.9		98.8		
9+	83.9		89.4		35.0		98.8		
10+	81.2		91.6		39.7		98.6		
**11+**	**79.2**	[60.3, 98.1]	**94.4**	[90.5, 98.3]	**49.0**	[29.4, 68.7]	**98.5**	[96.9, 1.0]	
12+	72.5		96.9		61.4		98.1		
13+	66.5		97.3		62.7		97.7		
ICD-10, any									
6+	98.0		77.1		22.8		99.8		0.959
7+	96.7		83.1		28.2		99.7		
8+	84.1		87.8		32.1		98.8		
9+	83.5		89.4		35.3		98.7		
10+	80.2		91.6		39.7		98.5		
**11+**	**78.2**	[59.6, 96.7]	**94.4**	[90.5, 98.3]	**49.0**	[29.4, 68.7]	**98.4**	[96.8, 1.0]	
12+	72.2		96.9		62.0		98.1		
13+	65.6		97.3		62.7		97.6		
ICD-10 moderate+severe									
6+	98.2		75.8		17.1		99.9		0.957
7+	97.3		81.7		21.3		99.8		
8+	82.3		86.4		23.5		99.0		
9+	82.3		88.1		26.0		99.0		
10+	82.3		90.5		30.5		99.0		
11+	82.3		93.4		38.6		99.0		
**12+**	**77.0**	[52.8, 1.0]	**96.0**	[93.4, 98.7]	**49.5**	[29.9, 69.1]	**98.8**	[97.2, 1.0]	
13+	72.6		96.6		51.9		98.6		

*Note.* PPV = positive predictive value; NPV = Negative predictive value; AUC = the area under the ROC curve; ICD-10 = International classification of mental and behavioural disorders 10th edition (WHO, 1992); DSM-5 = Diagnostic and Statistical Manual of Mental Disorders, 5th edition (American Psychiatric Association, 2013) CI = confidence Interval. Values in bold represent the most adequate combination of sensitivity, specificity, and PPV. For the sake of clarity 95% CIs provided only for the optimal cutoff-scores. 95% CIs for all values are reported in Table 3b which is provided by request to the first author. ^a^A value score exactly equal to the cutoff is understood as being depressed. ^b^’+’ signifies ‘or more’

For DSM-5 major depression as well as ICD-10 mild depression or more, a cutoff score of 11 or more is suggested by the data, both yielding a sensitivity close to 80%, a specificity above 90%, and a PPV close to 50%. For ICD-10 moderate and severe depression, a cutoff score of 12 or more was suggested with a sensitivity of 77%, specificity of 96% and a PPV of 49.5%.

As shown in Table 3, the ROC values between DSM-5 major and ICD-10 (any) are very similar. This is because only 10 women had a discrepant diagnostic status. However, the ROC values for ICD-10 moderate+severe are in some cases more different than DSM-5 because using this classification as ‘depressed’, and the ICD-10 mild as ‘not depressed’, resulted in 38 having a discrepant diagnostic status.

### Factor analysis

Using parallel analysis [54] we found that either two or three factors would be appropriate. To ensure a good fit we employ three factors, and the associated factor loadings are presented in Table 4. For clarity, only loadings of 0.3 and above are reported.

**Table 4:** Factor loadings in an EFA of the Danish EPDS

	Factor 1	Factor 2	Factor 3
% of variance	0.257	0.198	0.082
Item			
1 (anhedonia)	**0.888**		
2 (anhedonia)	**0.773**		
3 (guilt)		**0.605**	
4 (anxiety)		**0.805**	
5 (Panic attacks)		**0.748**	
6 (overwhelmed)	0.381	0.445	
7 (sleep problems)	0.357	0.309	
8 (sadness)	**0.701**		
9 (tearfulness)	**0.640**		
10 (self-harm and suicidal ideas)			**0.875**

*Note*. EFA: Exploratory Factor Analysis; only loadings of 0.300 or more are reported

Values in bold represent factor loadings included in either factor 1, 2, or 3

As shown in Table 4, items 6 and 7 cross loaded on factor 1 and factor 2. Likewise, the factor loadings of item 6 and 7 did not discriminate adequately to be included in either factor 1 or factor 2, and these were therefore omitted from both factors. Following, the result indicated that the Danish EPDS consists of three factors: Factor 1 (depression): items 1,2,8, and 9; Factor 2 (anxiety): items 3,4, and 5; and Factor 3 (self-harm/suicide): item 10. As shown, Factor 1 and Factor 2 are vastly more important than Factor 3 in terms of explained variance. It is noted that the third factor included essentially only loads on Item 10 (self-harm and suicidal ideas) and that this item did not load on the two other factors. For these reasons Factor 3 did not truly meet the criteria for being a factor as it is essentially just a rescaled version of item 10. We did, however, include it in this EFA, because it is important to realize that this item works in a different dimensionality from the rest.

## Discussion

In the EPDS literature, there is a wide variation as to what criteria have been used when the receiver operating characteristics of the scale have been assessed (i.e., the ‘gold standard’ has been defined by different diagnoses within and between different diagnostic systems). As previously suggested [16], we suspected that the variation in optimal cutoff scores reported across studies might at least partly be explained by this. Therefore, we validated the Danish EPDS against both DSM-5 and ICD-10 diagnostic criteria for depression.

In this Danish postnatal sample, for DSM-5 major as well as for ICD-10 mild, moderate or severe depression, a cutoff score of 11 or more was suggested by the data as the best cutoff. With sensitivities of 79.2 and 78.2%, specificities of 94.4%, and PPVs of 49% respectively, this value reflected the best trade-off in terms of the EPDS’s ability to detect the majority of cases of depression without an undue sacrifice of PPV.

Interestingly, four mothers met criteria for DSM-5 major depression but not ICD-10 mild depression (Table 2). This was due to the ICD-10 requirement of having at least two core symptoms out of three (as opposed to one of two in DSM-5), suggesting that there is an unfortunate inconsistency between the two diagnostic systems.

Because ICD-10 mild depression (which requires at least four versus five depressive symptoms in DSM-5 major depression) in clinical practice sometimes is considered “subthreshold”, for comparison, we also conducted analyses not including ICD-10 mild depression as ‘depressed’. In this case, and using our a priori defined ‘criteria’ for selecting cutoff scores, the optimal cutoff was 12 or more (sensitivity: 77.0%, specificity: 96%, PPV: 49.5%). However, given that the symptom threshold for ICD-10 moderate and severe depression is higher than for DSM-5 major depression (at least six symptoms in ICD-10 moderate versus at least five symptoms in DSM-5 Major), a missed case of moderate or severe depression is highly undesirable, and increasing sensitivity at the cost of PPV seems reasonable in this context. Hence, for the use in first-phase screening of Danish postnatal mothers, for ICD-10 moderate and severe depression, we recommend using a cutoff of 11 or more (sensitivity of 82.3%, specificity of 93.4%, and a PPV of 38.6%).

In sum, our results suggest that there are no notable differences with respect to using ICD-10 or DSM-5 criteria for depression in terms of optimal cutoff on the EPDS. Indeed, the ROC values are almost identical given that there were only 10 cases that showed discrepant caseness status. Thus, the optimum postpartum Danish cutoff (11 or more) differs from the English-speaking cutoff (13 or more for DSM major depression) as well as from a number of the cutoff scores found for other translations of the scales (e.g. [9, 10, 12, 14]). As also proposed previously [15], our results may suggest that the variation in cutoff scores across studies reflect differences in the expression of psychological distress across cultures, though, to our knowledge, no perinatal study have tested this assumption. More generally, our study stresses the importance of validating self-report instruments, originally validated in another culture (and another language), before use in a specific culture because cultural differences may impact results.

In the vast majority of EPDS validation studies, a diagnosis of depression has been used as the criterion or ‘gold standard’ against which the scale has been validated. This was also the case in our study where we used a thorough diagnostic interview. It should, however, be realized that some would argue that using such a criterion will miss many women who have significant levels of worry or low mood, yet do not meet diagnostic criteria for a mood disorder (e.g. [57–59]). Diagnostic status, therefore, may not be the most suitable criterion against which to validate mood screening instruments, but currently, this is the accepted methodology in the perinatal mental health field.

Another reason for the various cutoffs reported across studies could also be that, within the EPDS literature, there exist no agreed upon standards for what are acceptable levels of sensitivity, specificity, PPV and NPV. For screening purposes, high sensitivity is often desirable to ensure the detection of the majority of cases in the screened population. This was the case in the present study where we aimed at a sensitivity of 80% or more, a specificity of 90% or more, and a positive predictive value of 50% or more. However, high sensitivity is not always the first priority. For example, in a recent study, where a cutoff of 19 or more was selected, high specificity was prioritized and a sensitivity of 30% was considered acceptable for screening purposes in order to use the available resources in the most effective way and not overwhelm clinical services with many inappropriate referrals [60]. More generally, when using validated cutoff scores, the context in which the EPDS score is used is of crucial importance. For some research purposes, using an EPDS score as a measure of depression without further assessment (which is sometimes the case in epidemiological studies, e.g. [61]) it could be argued that the PPV should have a higher priority than sensitivity. This would ensure that those screening positive are very likely to meet diagnostic criteria for depression (if the PPV is, for example, 80% or 90%). In the current sample, if using a cutoff of 11 or more as a measure of depression, only approximately 5 out of every 10 screen positive mothers would, in fact, be depressed, which in turn would overestimate the prevalence of clinical depression in the population by a factor of two.

Using an exploratory factor analytic approach, our results indicate that the Danish EPDS is multidimensional as previously suggested. The first ‘depression factor’ included item 1 (anhedonia), item 2 (anhedonia), item 8 (sadness), and item 9 (tearfulness); the second ‘anxiety factor’ included item 3 (guilt), item 4 (anxiety), item 5 (panic attacks). As such, this result is in line with previous studies that have found an ‘anxiety factor’ of the EPDS to include items 3,4,5 [34, 35, 37, 38, 52, 62]. The anxiety subscale is sometimes referred to as the EPDS-3A or the EDS-3A in pregnancy [34, 63], and there is some evidence suggesting that it can be used to screen for perinatal anxiety [34, 52, 64]. However, more research is warranted to establish a separate cutoff score if this subscale should be used in clinical practice to screen for perinatal anxiety. Consistent with three previous studies [38, 39, 64], a third ‘self-harm/suicide factor’ (only including item 10) also emerged. However, in terms of proportion of explained variance, the two first factors were far more important than the third factor which accounted for less than 10% of the variance and fit statistics (parallel analysis) do not firmly establish whether the correct number of factors is two or three.

A limitation of the current study is that only women from urban Copenhagen area were included. This may limit generalizability to the whole population. Another limitation is that we did not have access to the number of mothers initially screened at T1 but who did not agree to be referred to the project, and thus we cannot report on the number of approached mothers who declined to participate. Neither are we able to report whether these mothers differ from our sample in terms of sociodemographic characteristics or EPDS scores. However, of the 350 mothers who were referred to the project, only 25 (7%) declined to participate. Yet, as these mothers did not give consent to participate, all of these mothers’ data were deleted, and therefore we were not able to report EPDS scores or sociodemographic characteristics on these mothers either. This problem, however, is very common within the EPDS literature, and to our knowledge, only two previous studies [25, 65] have reported on the number of approached women who declined to participate.

As we assessed depression according to the current diagnostic standards, we used the DSM-5 and ICD-10 diagnostic systems. Because the notion of minor depression (requiring two but less than five depressive symptoms) does not exist in DSM-5, this meant that the diagnostic interviews were not coded for DSM-IV minor depression. It could be argued that this would be relevant in terms of comparing our results with previous studies that have included women meeting criteria for minor depression as cases of depression and have reported cutoff scores for ‘combined depression’; and as such, this can be considered as a limitation of our study. Finally, when interpreting our findings the timeframe for T2 assessments should be considered. One of our inclusion criteria was that the mother had an infant between 2 and 10 months and although the majority (83%) of the T2 assessments were conducted between 8 and 20 weeks postpartum, T2 assessments were conducted with a quite wide timeframe (Table 1). Recently, a paper by Martin and Redshaw reported that mothers, who did not differ on other background variables, scored significantly different on the EPDS at three and six months postpartum [43]. However, based on the current data, it is not possible to address the question of whether an EPDS-score obtained for example at two months postpartum is more or less likely to reflect an underlying depression than at a later time point in the postpartum period.

Strengths of the study include the use of oversampling to ensure that a high number of depressed women were included, the sampling weighting method yielding good statistical power, and conducting interrater-reliability check of the clinicians who conducted the diagnostic interviews.

## Conclusions

The Danish EPDS is a valid and reliable screening instrument to detect possible depression in new mothers in a Danish postnatal population. The best cutoff score for the EPDS to screen for depression according to both DSM-5 and ICD-10 in Danish women is 11 or more. It should be noted, however, that the antenatal cutoff could be different, and possible, different for each trimester [15], and consequently, the scale should be validated in a antenatal sample before it is used to screen for depression in pregnancy. Moreover, the Danish EPDS is multi-dimensional, and, additional to measuring depressive symptoms and self-harm/suicidal ideas, it also contains a subscale measuring symptoms of anxiety. Thus, the appropriate validated cutoff score for this subscale would also need to be calculated for both the ante- and postnatal periods before being used to screen for perinatal anxiety.

## Additional files

Additional file 1: The Danish Edinburgh Postnatal Depression Scale (EPDS). (PDF 254 kb)

Additional file 2: Scoring sheet for the Danish EPDS. (PDF 35 kb)

## Abbreviations

AUC: Area under the ROC curve
AUC: Area under the ROC curve
CIMHP: Copenhagen Infant Mental Health Project
DSM-III-R: Diagnostic and Statistical Manual for Mental disorders, third edition, revised
DSM-5: Diagnostic and Statistical Manual for Mental disorders, fifth edition
DSM-IV-TR: Diagnostic and Statistical Manual for Mental disorders, fourth edition, revised
EFA: Exploratory factor analysis
EPDS: The Edinburgh Postnatal depression Scale
ICD-10: International Classification of Diseases 10th revision
NPV: Negative predictive value
PPV: Positive predictive value
ROCs: Receiver operating characteristics
SCID-5: The Structured Clinical Interview for the DSM-5
T1: Time 1
T2: Time 2

## References

[1] Cox JL, Holden JM, Sagovsky R (1987). Detection of postnatal depression. Development of the 10-item Edinburgh postnatal depression scale. Br J Psychiatry.

[2] Chaudron LH, Szilagyi PG, Kitzman HJ, Wadkins HI, Conwell Y (2004). Detection of postpartum depressive symptoms by screening at well-child visits. Pediatrics.

[3] Sheeder J, Kabir K, Stafford B (2009). Screening for postpartum depression at well-child visits: is once enough during the first 6 months of life?. Pediatrics.

[4] Hewitt CE, Gilbody SM, Mann R, Brealey S (2010). Instruments to identify post-natal depression: which methods have been the most extensively validated, in what setting and in which language?. Int J Psychiatry Clin Pract.

[5] Cox J, Holden J, Henshaw C: Perinatal mental health: the Edinburgh postnatal depression scale (EPDS) manual (2nd edn): RCPsych publications; 2014.

[6] Matthey S, Agostini F (2017). Using the Edinburgh postnatal depression scale for women and men-some cautionary thoughts. Archives of Women's Mental Health.

[7] Nielsen D, Videbech P, Hedegaard M, Dalby J, Secher N (2000). Postpartum depression: identification of women at risk. BJOG Int J Obstet Gynaecol.

[8] Mann R, Evans J. Screening tools and methods of identifying perinatal depression. Identifying Perinatal Depression and Anxiety: Evidence-based Practice in Screening, Psychosocial Assessment and Management. 2015;76.

[9] Bunevičius A, Kusminskas L, Bunevičius R (2009). Validation of the Lithuanian version of the Edinburgh postnatal depression scale. Medicina.

[10] Rowel D, Jayawardena P, Fernando N: Validation of the Sinhala translation of Edinburgh Postnatal Depression Scale. 2008. 10.4038/cmj.v53i1.219 18590263

[11] Guedeney N, Fermanian J (1998). Validation study of the French version of the Edinburgh postnatal depression scale (EPDS): new results about use and psychometric properties. European psychiatry.

[12] Wickberg B, Hwang C (1996). The Edinburgh postnatal depression scale: validation on a Swedish community sample. Acta Psychiatr Scand.

[13] Oates MR, Cox JL, Neema S, Asten P, Glangeaud-Freudenthal N, Figueiredo B, Gorman LL, Hacking S, Hirst E, Kammerer MH (2004). Postnatal depression across countries and cultures: a qualitative study. Br J Psychiatry.

[14] Barnett B, Matthey S, Gyaneshwar R (1999). Screening for postnatal depression in women of non-English speaking background. Archives of women's Mental Health.

[15] Kozinszky Z, Dudas RB (2015). Validation studies of the Edinburgh postnatal depression scale for the antenatal period. J Affect Disord.

[16] Gibson J, McKenzie-McHarg K, Shakespeare J, Price J, Gray R (2009). A systematic review of studies validating the Edinburgh postnatal depression scale in antepartum and postpartum women. Acta Psychiatr Scand.

[17] Garcia-Esteve Ls AC, Ojuel J, Navarro P (2003). Validation of the Edinburgh postnatal depression scale (EPDS) in Spanish mothers. J Affect Disord.

[18] Diagnostic and statistical manual of mental disorders fifth edition (DSM-5®): American Psychiatric Association; 2013.

[19] Diagnostic and statistical manual of mental disorders fourth edition DSM-IV-TR (2000). Washington.

[20] The ICD-10 classification of mental and behavioural disorders (1992). clinical descriptions and diagnostic guidelines.

[21] Cox JL, Chapman G, Murray D, Jones P (1996). Validation of the Edinburgh postnatal depression scale (EPDS) in non-postnatal women. J Affect Disord.

[22] Spitzer R, Endicott J, Robins E (1975). Research diagnostic criteria: instrument no. 58.

[23] Alvarado-Esquivel C, Sifuentes-Alvarez A, Salas-Martinez C, Martínez-García S (2006). Validation of the Edinburgh postpartum depression scale in a population of puerperal women in Mexico. Clin Pract Epidemiol Ment Health.

[24] Aydin N, Inandi T, Yigit A, Hodoglugil NNS (2004). Validation of the Turkish version of the Edinburgh postnatal depression scale among women within their first postpartum year. Soc Psychiatry Psychiatr Epidemiol.

[25] Töreki A, Andó B, Keresztúri A, Sikovanyecz J, Dudas RB, Janka Z, Kozinszky Z, Pál A (2013). The Edinburgh postnatal depression scale: translation and antepartum validation for a Hungarian sample. Midwifery.

[26] First MB (1997). User's guide for the structured clinical interview for DSM-IV axis I disorders SCID-I: clinician version.

[27] Sheehan D, Janavs J, Baker R, Harnett-Sheehan K, Knapp E, Sheehan M, Lecrubier Y, Weiller E, Hergueta T, Amorim P (1998). MINI-Mini international neuropsychiatric interview-english version 5.0. 0-DSM-IV. J Clin Psychiatry.

[28] Jenkins R, Goldberg D, Kiima D, Mayeya J, Mayeya P, Mbatia J, Mussa M, Njenga F, Okonji M, Paton J (2002). Classification in primary care: experience with current diagnostic systems. Psychopathology.

[29] Mezzich JE (2002). International surveys on the use of ICD-10 and related diagnostic systems. Psychopathology.

[30] Bowen A, Bowen R, Maslany G, Muhajarine N (2008). Anxiety in a socially high-risk sample of pregnant women in Canada. Can J Psychiatry.

[31] Matthey S, Fisher J, Rowe H (2013). Using the Edinburgh postnatal depression scale to screen for anxiety disorders: conceptual and methodological considerations. J Affect Disord.

[32] Heron J, O'Connor TG, Evans J, Golding J, Glover V, Team AS (2004). The course of anxiety and depression through pregnancy and the postpartum in a community sample. J Affect Disord.

[33] Kingston D, Tough S, Whitfield H (2012). Prenatal and postpartum maternal psychological distress and infant development: a systematic review. Child Psychiatry Hum Dev.

[34] Matthey S (2008). Using the Edinburgh postnatal depression scale to screen for anxiety disorders. Depression and anxiety.

[35] Pop VJ, Komproe IH, Van Son MJ (1992). Characteristics of the Edinburgh post natal depression scale in the Netherlands. J Affect Disord.

[36] King PAL (2012). Replicability of structural models of the Edinburgh postnatal depression scale (EPDS) in a community sample of postpartum African American women with low socioeconomic status. Archives of women's mental health.

[37] Kubota C, Okada T, Aleksic B, Nakamura Y, Kunimoto S, Morikawa M, Shiino T, Tamaji A, Ohoka H, Banno N (2014). Factor structure of the Japanese version of the Edinburgh postnatal depression scale in the postpartum period. PLoS One.

[38] Jomeen J, Martin CR (2007). Replicability and stability of the multidimensional model of the Edinburgh postnatal depression scale in late pregnancy. J Psychiatr Ment Health Nurs.

[39] Small R, Lumley J, Yelland J, Brown S (2007). The performance of the Edinburgh postnatal depression scale in English speaking and non-English speaking populations in Australia. Soc Psychiatry Psychiatr Epidemiol.

[40] Kozinszky Z, Töreki A, Hompoth EA, Dudas RB, Németh G (2017). A more rational, theory-driven approach to analysing the factor structure of the Edinburgh postnatal depression scale. Psychiatry Res.

[41] Petrozzi A, Gagliardi L (2013). Anxious and depressive components of Edinburgh postnatal depression scale in maternal postpartum psychological problems1. J Perinat Med.

[42] Montazeri A, Torkan B, Omidvari S (2007). The Edinburgh postnatal depression scale (EPDS): translation and validation study of the Iranian version. BMC psychiatry.

[43] Martin CR, Redshaw M (2018). Establishing a coherent and replicable measurement model of the Edinburgh postnatal depression scale. Psychiatry Res.

[44] Gollan JK, Wisniewski SR, Luther JF, Eng HF, Dills JL, Sit D, Ciolino JD, Wisner KL (2017). Generating an efficient version of the Edinburgh postnatal depression scale in an urban obstetrical population. J Affect Disord.

[45] Depression in adults: recognition and management. Clinical guideline [CG90]. [https://www.nice.org.uk/guidance/cg90].

[46] Væver MS, Smith-Nielsen J, Lange T (2016). Copenhagen infant mental health project: study protocol for a randomized controlled trial comparing circle of security–parenting and care as usual as interventions targeting infant mental health risks. BMC psychology.

[47] Edmondson OJ, Psychogiou L, Vlachos H, Netsi E, Ramchandani PG (2010). Depression in fathers in the postnatal period: assessment of the Edinburgh postnatal depression scale as a screening measure. J Affect Disord.

[48] Dunn G, Pickles A, Tansella M, Vázquez-Barquero JL. Two-phase epidemiological surveys in psychiatric research. Br J Psychiatry. 1999. 10.1192/bjp.174.2.95 10211161

[49] Lasa L, Ayuso-Mateos J, Vazquez-Barquero J, Dıez-Manrique F, Dowrick C (2000). The use of the Beck depression inventory to screen for depression in the general population: a preliminary analysis. J Affect Disord.

[50] Cochran WG (1977). Sampling techniques. In*.*, 3rd edn.

[51] First M, Williams J, Karg R, Spitzer R (2015). Structured clinical interview for DSM-5—research version (SCID-5 for DSM-5, research version; SCID-5-RV).

[52] Matthey S, Ross-Hamid C (2011). The validity of DSM symptoms for depression and anxiety disorders during pregnancy. J Affect Disord.

[53] Fleiss JL, Levin B, Paik MC. Statistical methods for rates and proportions: John Wiley & Sons; 2013.

[54] Revelle WR. Psych: procedures for personality and. Psychol Res. 2017.

[55] Watson D (2005). Rethinking the mood and anxiety disorders: a quantitative hierarchical model for DSM-V. J Abnorm Psychol.

[56] Tavakol M, Dennick R (2011). Making sense of Cronbach's alpha. Int J Med Educ.

[57] Tyrer P (2014). A comparison of DSM and ICD classifications of mental disorder. Adv Psychiatr Treat.

[58] Green JM (1998). Postnatal depression or perinatal dysphoria? Findings from a longitudinal community-based study using the Edinburgh postnatal depression scale. Journal of Reproductive and Infant Psychology.

[59] Goodman JH, Tyer-Viola L (2010). Detection, treatment, and referral of perinatal depression and anxiety by obstetrical providers. J Women's Health.

[60] van der Westhuizen C, Brittain K, Koen N, Maré K, Zar HJ, Stein DJ. Sensitivity and specificity of the SRQ-20 and the EPDS in diagnosing major depression ante-and postnatally in a south African birth cohort study. Int J Ment Heal Addict. 2017:1–12.

[61] Tharner A, Luijk MP, Van IJzendoorn MH, Bakermans-Kranenburg MJ, Jaddoe VW, Hofman A, Verhulst FC, Tiemeier H (2012). Maternal lifetime history of depression and depressive symptoms in the prenatal and early postnatal period do not predict infant–mother attachment quality in a large, population-based Dutch cohort study. Attach Hum Dev.

[62] Brouwers EPM, van Baar AL, Pop VJM (2001). Does the Edinburgh postnatal depression scale measure anxiety?. J Psychosom Res.

[63] Ayers S, Coates R, Matthey S. Identifying perinatal anxiety. Identifying Perinatal Depression and Anxiety: Evidence-Based Practice in Screening, Psychosocial Assessment, and Management. 2015:93–107.

[64] Ross LE, Evans SG, Sellers E, Romach M (2003). Measurement issues in postpartum depression part 1: anxiety as a feature of postpartum depression. Archives of Women's Mental Health.

[65] Rubertsson C, Börjesson K, Berglund A, Josefsson A, Sydsjö G (2011). The Swedish validation of Edinburgh postnatal depression scale (EPDS) during pregnancy. Nordic journal of psychiatry.

