# Supplementary material for: Validation of the Edinburgh Postnatal Depression Scale against both DSM-5 and ICD-10 diagnostic criteria for depression
Source: BMC Psychiatry. 2018 Dec 20;18:393. doi: 10.1186/s12888-018-1965-7 (PMC6302501; doi:10.1186/s12888-018-1965-7)
Supplement: Supplementary file 1 — The Danish Edinburgh Postnatal Depression Scale (EPDS). (PDF 254 kb) [file 12888_2018_1965_MOESM1_ESM.pdf]

Edinburgh Postnatal Depression Scale

Hvordan har du det?

Nu, hvor du har fået et barn, vil vi gerne vide, hvordan du har haft det inden for de sidste 7 dage. Vær venlig at <u>understrege</u> det svar, som du synes bedst beskriver, hvordan du har haft det i løbet af de sidste 7 dage - og altså ikke blot i dag. Her er et eksempel, som allerede er udfyldt:

Jeg har følt mig lykkelig:

Ja, det meste af tiden
<u>Ja, noget af tiden</u>
Nej, ikke særlig ofte
Nej, slet ikke

Dette svar betyder: "jeg har følt mig lykkelig noget af tiden den seneste uge". Vær venlig at udfylde de nedenstående spørgsmål på samme måde.

I de sidste 7 dage:

1. Har jeg kunnet le og se tingene fra den humoristiske side
   Lige så meget som jeg altid har kunnet
   Ikke helt så meget som tidligere
   Afgjort ikke så meget som tidligere
   Overhovedet ikke

2. Har jeg kunnet se frem til ting med glæde
   Lige så meget som jeg tidligere har gjort
   En del mindre end jeg tidligere har gjort
   Afgjort mindre end jeg tidligere har gjort
   Næsten ikke

3. Har jeg unødvendigt bebrejdet mig selv, når ting ikke gik som de skulle
   Ja, det meste af tiden
   Ja, af og til
   Nej, sjældent
   Nej, aldrig

4. Har jeg været anspændt og bekymret uden nogen særlig grund
   Nej, overhovedet ikke
   Meget sjældent
   Ja, nogle gange
   Ja, meget ofte

5. Har jeg følt mig angst eller panikslagen uden nogen særlig grund
Ja, en hel del
Ja, nogle gange
Nej, ikke meget
Nej, overhovedet ikke

6. Har jeg følt, at tingene voksede mig over hovedet
Ja, det meste af tiden
Ja, nogle gange
Nej, det meste af tiden har jeg kunnet overskue min situation
Nej, jeg har kunnet overskue min situation lige så godt, som jeg plejer

7. Har jeg været så ked af det, at jeg har haft svært ved at sove
Ja, det meste af tiden
Ja, nogle gange
Nej, sjældent
Nej, aldrig

8. Har jeg følt mig trist eller elendigt til mode
Ja, det meste af tiden
Ja, ret tit
Nej, sjældent
Nej, aldrig

9. Har jeg været så ulykkelig, at jeg har grædt
Ja, det meste af tiden
Ja, ret tit
Nej, kun ved enkelte lejligheder
Nej, aldrig

10. Har jeg tænkt på at gøre skade på mig selv
Ja, ganske ofte
Nogle gange
Meget sjældent
Aldrig

Original version:
Cox, J. L., Holden, J. M., & Sagovsky, R. (1987). Detection of Postnatal Depression - Development of the 10-Item Edinburgh Postnatal Depression Scale. *British Journal of Psychiatry, 150,* 782-786

Dansk version:
Denne version er en modificeret ved Smith-Nielsen, J., & Væver, M.S., Københavns Universitets BabyLab (2015) fra Dansk oversættelse af: Nielsen D, Videbech P, Hedegaard M, Dalby J, Secher NJ: Postpartum depression: identification of women at risk. BJOG: An International Journal of Obstetrics & Gynaecology 2000;107:1210-1217.
