# Supplementary material for: Validation of the Edinburgh Postnatal Depression Scale against both DSM-5 and ICD-10 diagnostic criteria for depression
Source: BMC Psychiatry. 2018 Dec 20;18:393. doi: 10.1186/s12888-018-1965-7 (PMC6302501; doi:10.1186/s12888-018-1965-7)
Supplement: Supplementary file 2 — Scoring sheet for the Danish EPDS. (PDF 35 kb) [file 12888_2018_1965_MOESM2_ESM.pdf]

Edinburgh Postnatal Depression Scale: Scoringsark

NB! Dette er et scoringsark og skal <u>ikke</u> udfyldes af forælderen, men bruges af sundhedsplejersken til at give en score på det udfyldte spørgeskema.

1. Har jeg kunnet le og se tingene fra den humoristiske side

Lige så meget som jeg altid har kunnet	0
Ikke helt så meget som tidligere	1
Afgjort ikke så meget som tidligere	2
Overhovedet ikke	3

2. Har jeg kunnet se frem til ting med glæde

Lige så meget som jeg tidligere har gjort	0
En del mindre end jeg tidligere har gjort	1
Afgjort mindre end jeg tidligere har gjort	2
Næsten ikke	3

3. Har jeg unødvendigt bebrejdet mig selv, når ting ikke gik som de skulle

Ja, det meste af tiden	3
Ja, af og til	2
Nej, sjældent	1
Nej, aldrig	0

4. Har jeg været anspændt og bekymret uden nogen særlig grund

Nej, overhovedet ikke	0
Meget sjældent	1
Ja, nogle gange	2
Ja, meget ofte	3

5. Har jeg følt mig angst eller panikslagen uden nogen særlig grund

Ja, en hel del	3
Ja, nogle gange	2
Nej, ikke meget	1
Nej, overhovedet ikke	0

6. Har jeg følt at tingene voksede mig over hovedet

Ja, det meste af tiden	3
Ja, nogle gange	2
Nej, det meste af tiden har jeg kunnet overskue min situation	1
Nej, jeg har kunnet overskue min situation lige så godt, som jeg plejer	0

7. Har jeg været så ked af det, at jeg har haft svært ved at sove

Ja, det meste af tiden	3
Ja, nogle gange	2
Nej, sjældent	1
Nej, aldrig	0

8. Har jeg følt mig trist eller elendigt til mode

Ja, det meste af tiden	3
Ja, ret tit	2
Nej, sjældent	1
Nej, aldrig	0

9. Har jeg været så ulykkelig, at jeg har grædt

Ja, det meste af tiden	3
Ja, ret tit	2
Nej, kun ved enkelte lejligheder	1
Nej, aldrig	0

10. Har jeg tænkt på at gøre skade på mig selv

Ja, ganske ofte	3
Nogle gange	2
Meget sjældent	1
Aldrig	0
